# Trends in level of education and area of residence of users of a mobile app to support treatment of urinary incontinence

**DOI:** 10.1186/s12913-025-12632-w

**Published:** 2025-04-03

**Authors:** Ina Asklund, Stina Åhman, Anna Lindam, Eva Samuelsson

**Affiliations:** 1 Department of Public Health and Clinical Medicine, Umeå University, Umeå, Sweden; 2 Department of Public Health and Clinical Medicine, Unit of Research, Education, and Development-Östersund, Umeå University, Umeå, Sweden

**Keywords:** Mobile apps, Pelvic floor muscle training, Urinary incontinence, Education, Residence, Demographics, Socioeconomics

## Abstract

**Background:**

Between 25% and 45% of women experience urinary incontinence. The Tät^®^ app is intended to treat stress urinary incontinence in women, and has been evaluated for efficacy and effectiveness. The level of usage of digital healthcare differs depending on demographics, and this might lead to unequal access to healthcare. This study aims to analyse the change in level of education and area of residence of users of the Tät app over time, and compare this with overall demographic data for Sweden.

**Methods:**

When the app was downloaded, the user was invited to respond anonymously to a questionnaire. We included women aged 18–89 years living in Sweden. We weighted the app data to reflect the age distribution of the general female population of Sweden. We then compared the users’ level of education and area of residence with data from Statistics Sweden.

**Results:**

The study encompassed 153,819 users between 2016 and 2021. The percentage of Tät users with university education decreased from 63.14% (95% CI 62.16–64.11) to 61.07% (95% CI 60.53–61.61), and the percentage of users with fewer than 7 years of education increased from 0.02% (95% CI 0.006–0.077) to 1.94% (95% CI 1.80–2.10). In contrast to this, the Statistics Sweden data comparing 2016 with 2020, showed an increase in the category “university or higher education institution” from 38.94 to 42.10% and a decrease in the other categories. Comparing Tät users’ area of residence between 2018 and 2021 showed an increase in the amount of users living in rural areas from 16.90% (95% CI 16.44–17.37) to 20.53% (95% CI 20.08–20.98). Data from Statistics Sweden did not show any significant change, and in 2020 6.23% of women in Sweden lived in rural areas.

**Conclusions:**

The proportion of Tät users in both the highest and the lowest educational categories had changed to be more like the overall Swedish female population. The proportion of Tät users living in rural areas had increased and was considerably larger than for the population in general. We thus see positive trends in the distribution of users, although users with a university education are still over-represented.

## Background

Studies on the occurrence of urinary incontinence (UI) show that 25–45% of the female population experience some type of UI during their lifetime, to varying degrees and in different time-spans [1]. There are several types of UI: stress urinary incontinence (SUI), urgency urinary incontinence (UUI) and mixed urinary incontinence (MUI), which is a combination of the first two [2]. SUI is the most common type [1, 3].

Women who experience UI attest to it having a distinctly negative effect on their perceived quality of life with an increasing impact corresponding to the amount of urinary leakage [4, 5]. Women do not always seek help for UI, for a range of reasons including not thinking of UI as a disease, shame, negative support from their social networks or shortcomings in the healthcare system [6]. A person’s socioeconomic status correlates with their general knowledge about UI [7].

The recommended first-line treatment for UI is pelvic floor muscle training (PFMT) and lifestyle interventions. This combination has been shown to provide effective treatment for UI with a subsequent positive effect on the quality of life in terms of social functioning [8, 9]. Studies have shown that PFMT cures or improves symptoms in 74% of women with SUI and that it can also have a positive effect on other types of UI [10].

Tät^®^ is a smartphone app designed to be an easily accessible tool for treating SUI via PFMT and lifestyle advice. The efficacy of the Tät program among women has been evaluated in a randomized controlled trial. Women who used the Tät app experienced significantly improved UI symptoms and quality of life after three months’ use, and the number of leakages decreased in comparison with controls [11]. The Tät app was also perceived as an easily accessible and supportive tool for performing an unsupervised PFMT program [12]. Moreover, the app has been shown to be cost-efficient and also effective in real-world use [13, 14].

One strength of app-based training tools is the broad use of smartphones, which has increased considerably over the years. In 2011, 27% of the Swedish population owned a smartphone. In 2019 the percentage had increased to 92% [15], compared to 67% smartphone ownership worldwide in 2020 [16]. However, there are surveys that indicate limitations in app-based healthcare. One problem is that the elderly, people with lower income, and people with a lower level of education use e-health solutions less [17, 18]. Also, in Sweden there is a difference in the level of education of people who use digital healthcare services, with a lower usage in the group with the lowest level of education [15]. Moreover, previous studies of the use of the Tät app showed that users were predominantly highly educated [19–21].

The aim of this study was to analyse whether the profile of users of the Tät app changed over time in terms of level of education and area of residence, and the extent to which this corresponds with the distribution of these factors in the general Swedish population.

## Methods

This cohort study analysed the usage of the Tät^®^ app in the real world. The app was made freely available to the public on App Store and Google Play from June 2015 and a questionnaire has always been included as part of the download procedure. There was no specific marketing campaign for the app during the study. However, the app and the research behind the app is acknowledged by health care professionals in Sweden, and thus some of the users that turned to ordinary care, might have got recommendations to use it. There was an update to the questionnaire in January 2018 with the addition of questions regarding the user’s area of residence. More details on the questionnaire can be found in the article by Löjdahl et al. [22].

**Fig. 1:**
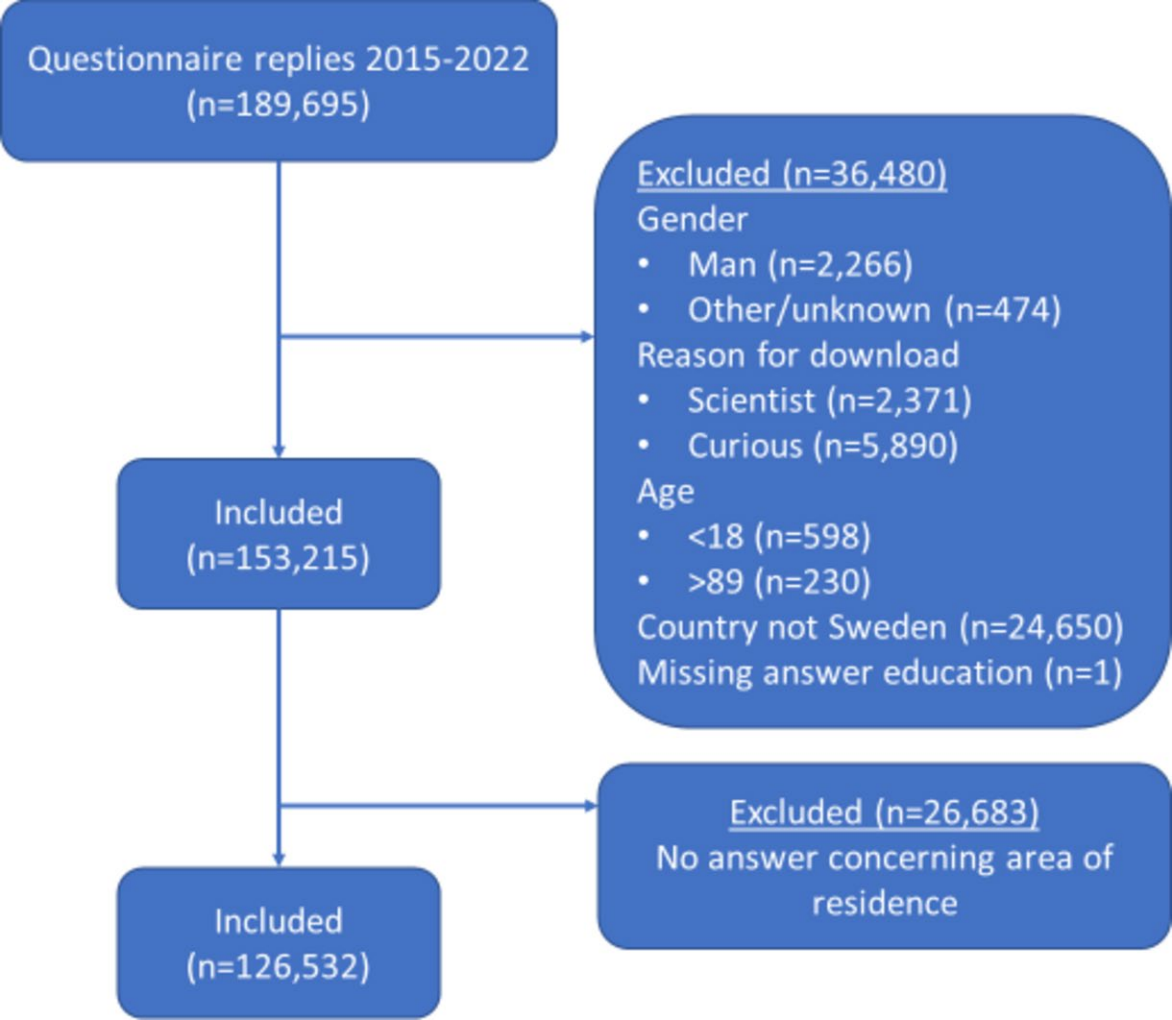
Flowchart of the number of participants included/excluded in the analyses of Tät^®^

This study included users aged 18–89 years, living in Sweden, who stated that they downloaded the app to treat urinary incontinence or to use as prevention for it. We included users who stated that they were female. However, before January 2018 no questions were asked about gender, reasons for downloading the app or the country of residence of the user. Before January 2018, the app was only available in Swedish and English. Therefore, all users prior to the 2018 update were included in the analyses of the Tät users’ level of education, if the other criteria were met (Fig. 1).

When they used the app for the first time, users were informed about the study and invited to complete a voluntary questionnaire anonymously which contained questions regarding demographics, reasons for using the app and symptoms of UI. The responses were sent automatically to an encrypted research database and no data was stored in the app. The answers could not be traced back to the user, and no information about their name, social security number, e-mail, telephone number, IMEI code or IP address was asked for. As such, no identifying personal data were collected for this study.

When replying to the question about their level of education, the users were offered the following alternatives: six years of education or less, elementary school or 7–9 years of education, upper-secondary education or 10–12 years of education, university or other higher education institution. The response alternatives available for area of residence were the following: in a rural area, in a district with fewer than 50,000 residents, in a district with 50,000–1,000,000 residents, in a city with more than one million residents.

**Fig. 2:**
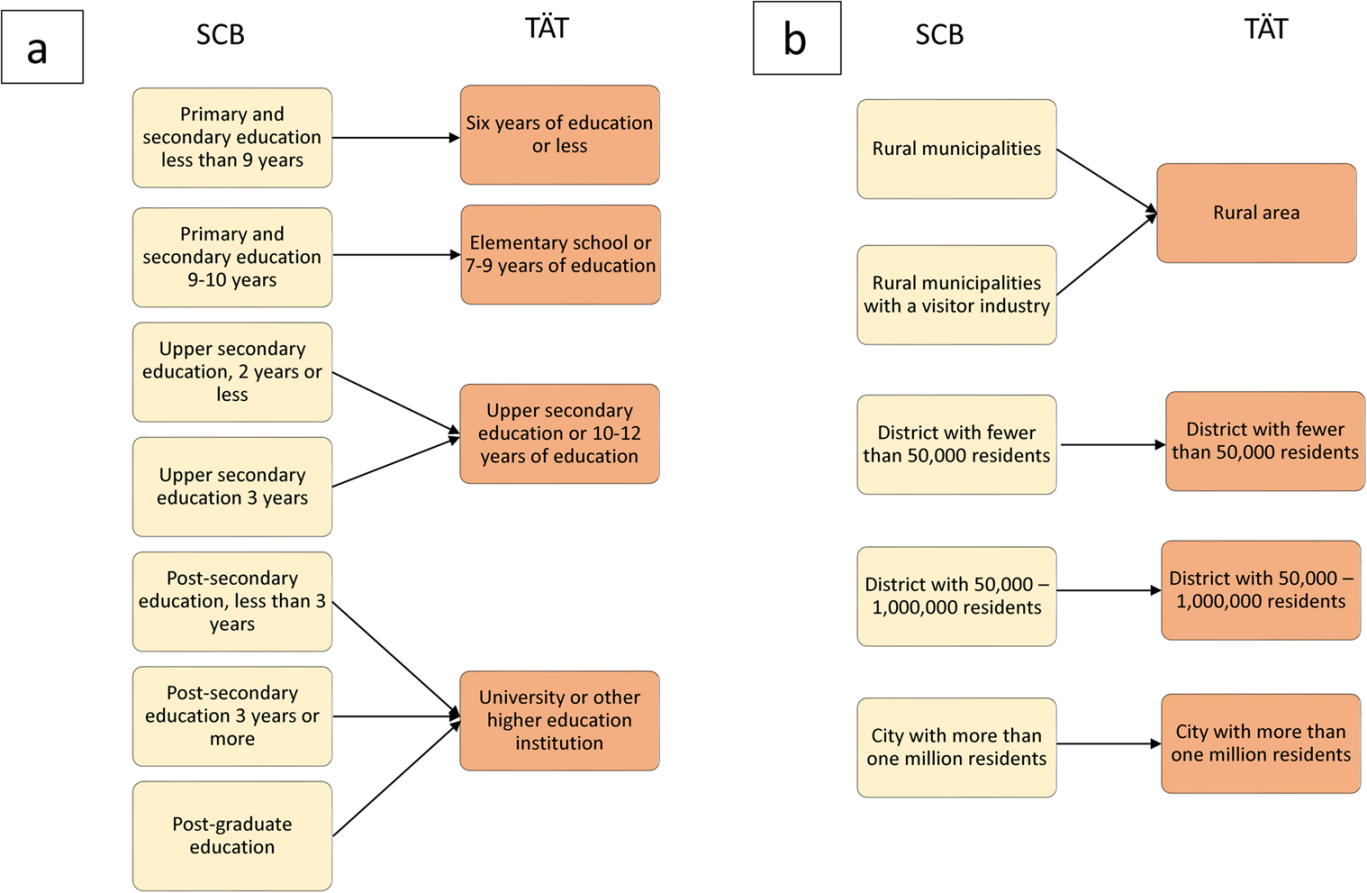
**a**, **b** The categorization of level of education and area of residence of the Swedish female population (SCB data) grouped in accordance with the Tät data

The users were categorized by age and year of response to the questionnaire and were then compared to data for the entire female population of Sweden from Statistics Sweden (SCB). The SCB data had seven different categories of educational level whereas the Tät data only had four. The categories nevertheless matched with overlaps in most cases, with the exception of the group with the lowest level of education, for which SCB used “eight years of education or less” compared with Tät’s “six years of education or less” (Fig. 2).

The categorization of area of residence also differed between the SCB and Tät data, so the SCB data was therefore grouped in accordance with the Tät data to enable comparison. The SCB’s population data was categorized according to the Swedish Association of Local Authorities and Regions’ (SKR) “Classification of Swedish municipalities 2017”. The SKR categories C8 (Rural municipalities) and C9 (Rural municipalities with a visitor industry) were combined to correspond to the category of “rural area” in the Tät data. The remaining Tät categories were compared to the population distribution across Swedish municipalities (Fig. 2). Some Tät users reported that they live in a city with more than one million inhabitants, while the SCB data indicates that there are no such large cities in Sweden. At the time of the study, the SCB only held data for up until 2020. As the change in area of residence was negligible between 2015 and 2020, this study presumed that there were no major shifts in the same categories between 2021 and 2022 to allow comparisons to be made.

### Statistical analysis

**Fig. 3:**
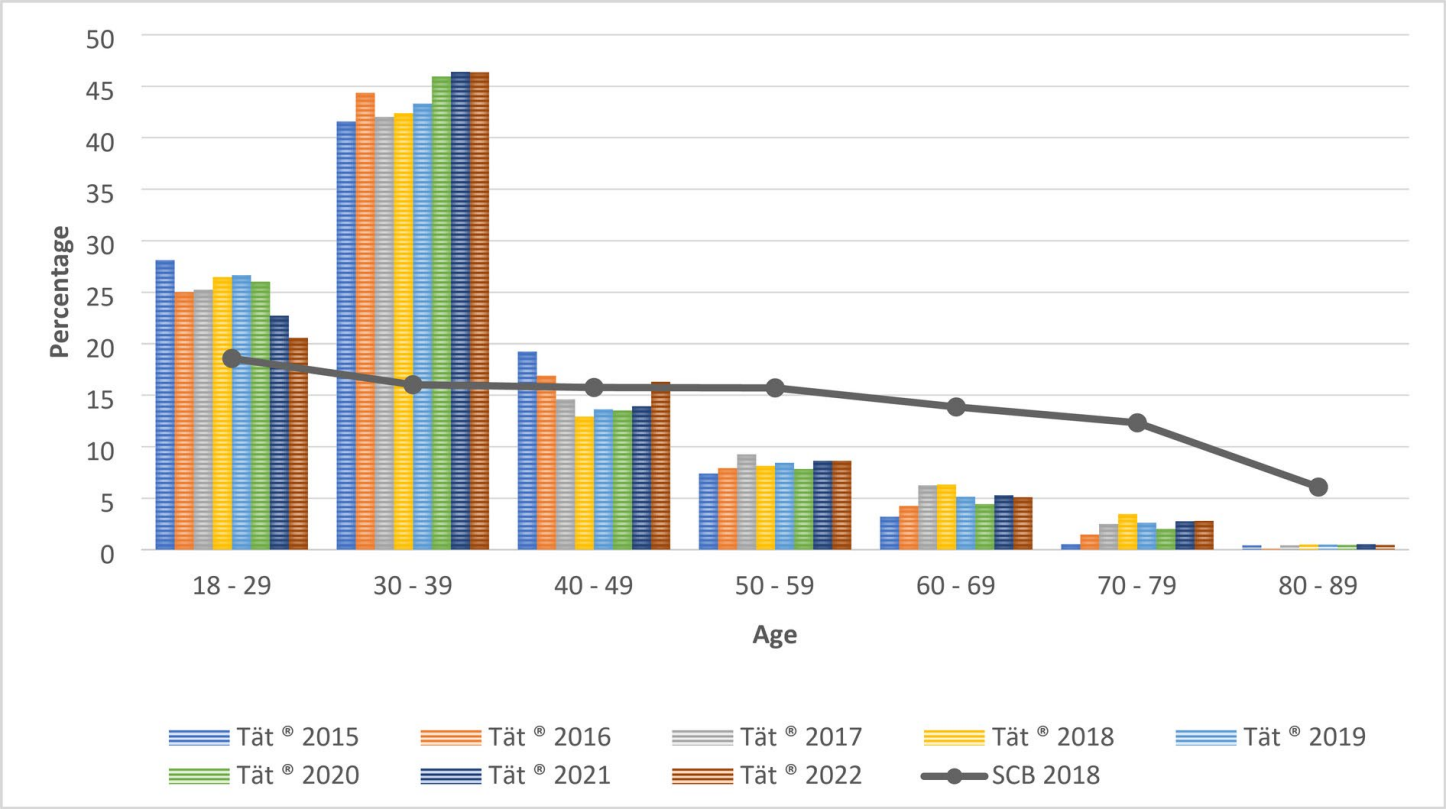
Age distribution Tät^®^ (2015–2022) and SCB (2018) in percentage

As expected, there was a discrepancy in the age distribution between the Tät data and the Swedish female population (SCB data) as the use of the app is prevalent during pregnancy and in middle-aged women with a high prevalence of urinary incontinence (Fig. 3).

As we know that there is an association between age, level of education and area of residence in Sweden, it is possible that differences seen in the level of education and area of residence are confounded by age differences. To be able to compare the Tät data with SCB data, the Tät data was therefore weighted to reflect the age distribution of the overall Swedish population in the SCB data. The weighting was created by dividing the number of people in each age category for each year by the total number of people of the same year. The ratio was then divided by the ratio for the same age category obtained from the SCB data in a similar manner. The Tät data was weighted according to SCB’s 2018 data on age distribution of women aged 18–89 years. This method was deemed acceptable since the last ten years of SCB data shows there has been little change in age distribution.

A confidence interval (Wilson score) was used to illustrate the difference between years to establish the significance of the results. The change was considered significant if there was no overlap in the confidence interval between the different years within the same category. The data was analysed using IBM^®^ SPSS^®^ statistics 28 and Microsoft^®^ Excel.

### Ethics

All data used in this study is anonymous. Before answering the questionnaire, the users were asked to tick a box to confirm that they had read the information about the study. The study was approved by the Swedish Ethical Review Authority (Dnr 2023-00352-02, 2014-389-32 M, 2016-80-32 M, 2017-405-32 M added to 2012-325-31 M).

## Results

For the analyses of the level of education, 153,215 users were included from the Tät app and for the area of residence 126,532 users were included (Fig. 1). The mean age was 37 years. Figure 3 shows the age distribution over time (Fig. 3).

### Tät, level of education

The analysis of the Tät data and comparison between 2015 and 2022 showed that there had only been a significant change in the category “six years of education or less”. However, data was not collected for all months of either 2015 or 2022. A comparison of 2016 and 2021, both of which had a substantially larger quantity of data, shows a statistically significant change in all groups. Between 2016 and 2021 the percentage of Tät users with a university education decreased from 63.14% (95% CI 62.16–64.11) to 61.07% (95% CI 60.53–61.61), and the percentage of users with six years of education or less increased from 0.02% (95% CI 0.006–0.077) to 1.94% (95% CI 1.80–2.10) (Table 1).

**Table 1:** Level of education in percentage for women using Tät^®^ (weighted data) and for the average Swedish female population (SCB data). For the Tät data the number of users is included, as well as percentages and confidence intervals. The SCB data includes the entire population, therefore no confidence intervals are calculated

Level of education	Data set	2015 Tät^®^*n* = 2,415	2016 Tät^®^*n* = 9,438	2017 Tät^®^*n* = 14,830	2018 Tät^®^*n* = 2,5316	2019 Tät^®^*n* = 34,192	2020 Tät^®^*n* = 33,109	2021 Tät^®^*n* = 31,226	2022 Tät^®^*n* = 2,689
Six years of education or less % (95%CI)	Tät^®^	0 (0.00–1.16)	0.02 (0.006–0.077)	0.03 (0.01–0.07)	2.16 (1.99–2.35)	2.16 (2.01–2.32)	1.88 (1.74–2.03)	1.94 (1.80–2.10)	2.83 (2.26–3.52)
	SCB*	9.19	8.71	8.24	7.80	7.38	6.93	No data	No data
Elementary school/7–9 years of education % (95%CI)	Tät^®^	5.92 (5.05–6.93)	7.69 (7.17–8.25)	6.62 (6.23–7.03)	5.53 (5.26–5.82)	6.71 (6.45–6.99)	5.47 (5.23–5.72)	5.59 (5.34–5.85)	4.35 (3.643–5.19)
	SCB	9.13	9.01	8.90	8.82	8.73	8.67	No data	No data
Upper-secondary education/10–12 years of education % (95%CI)	Tät^®^	28.16 (26.40–29.98)	29.15 (28.24–30.07)	32.32 (31.57–33.08)	32.32 (31.75–32.90)	32.54 (32.05–33.04)	31.54 (31.04–32.04)	31.39 (30.88–31.91)	30.42 (28.71–32.19)
	SCB	41.79	41.46	41.22	40.86	40.52	40.27	No data	No data
University or higher education institution % (95% CI)	Tät^®^	65.92 (64.01–67.78)	63.14 (62.16–64.11)	61.03 (60.24–61.81)	59.98 (59.48–60.58)	58.58 (58.06–59.10)	61.11 (60.58–61.63)	61.07 (60.53–61.61)	62.40 (60.58–64.2)
	SCB	38.17	38.94	39.66	40.47	41.24	42.10	No data	No data

*n* number of users

*SCB category: eight years of education or less

In contrast, in the data obtained from SCB comparing 2016 with 2020, there was an increase from 38.94 to 42.10% in the SCB category “university education” and a decrease in the other categories.

The comparison of the Tät data and SCB data for 2016 and 2020 showed greater resemblance in all categories, with the exception of “elementary school/7–9 years of education” (Table 1).

### Tät, area of residence

Comparing 2018 to 2022 showed that the percentage of Tät users living in rural areas increased significantly from 16.90% (95% CI 16.44–17.37) to 21.31% (95% CI 19.8–22.9). There was also a statistically significant decrease in the category “district with fewer than 50,000 inhabitants” (Table 2).

**Table 2:** Area of residence in percentage for women using Tät^®^ (weighted data) and for the average Swedish female population (SCB data). For the Tät data the number of users is included, as well as percentages and confidence intervals. The SCB data includes the entire population, therefore no confidence intervals are calculated

Area of residence	Data set	2018 Tät^®^*n* = 25,316	2019 Tät^®^*n* = 34,192	2020 Tät^®^*n* = 33,109	2021 Tät^®^*n* = 31,226	2022 Tät^®^*n* = 2,689
Rural area % (95% CI)	Tät^®^	16.90 (16.44–17.37)	18.04 (17.64–18.45)	19.77 (19.34–20.2)	20.53 (20.08–20.98)	21.31 (19.8–22.9)
	SCB	6.37	6.29	6.23	No data	No data
District with fewer than 50,000 inhabitants % (95% CI)	Tät^®^	28.59 (28.03–29.15)	28.43 (27.96–28.91)	27.29 (26.81–27.77)	27.05 (26.56–27.55)	25.44 (23.83–27.12)
	SCB	35.53	35.42	35.39	No data	No data
District with 50,000–1 million inhabitants % (95% CI)	Tät^®^	36.47 (35.88–37.07)	36.22 (35.72–36.74)	36.04 (35.53–36.56)	34.53 (34–35.05)	36.44 (34.65–38.28)
	SCB	58.10	58.30	58.38	No data	No data
City with more than 1 million inhabitants % (95% CI)	Tät^®^	18.04 (17.57–18.52)	17.30 (16.9–17.7)	16.90 (16.5–17.31)	17.89 (17.47–18.32)	16.81 (15.44–18.29)
	SCB	not applicable	not applicable	not applicable	not applicable	not applicable

*n* number of users

In 2018, the proportion of Tät users living in rural areas was over-represented (Tät 16.90%) compared to the overall Swedish population (SCB 6.37%), with a gap of 10.53% points (pp). Given the presumption that the SCB data remained proportionally constant between 2020 and 2021, this difference increased further to 14.30 pp in 2021.

## Discussion

In this large cohort study of over 150,000 app users we found that users with a high level of education were still over-represented, compared to the general Swedish population. However, the changes in usage that are seen over a 5-year period indicate that the disparity with the overall Swedish population decreases over time. We also found that users in rural areas were over-represented, and even growing.

The distribution of the Tät users in terms of level of education became more consistent with the SCB data for all educational categories apart from “elementary school/7–9 years of education” between 2016 and 2020. However, there was still a larger proportion of highly educated women using the app, and there still seems to be a digital divide with people with less education being less likely to use digital technology [15].

The comparison between the Tät users’ areas of residence and the SCB data is somewhat lacking due to mismatched categories. What can nevertheless be seen is a change in the Tät data in that the proportion of users in rural areas increased from 2018 to both 2021 and 2022. This result is consistent with the survey “The Swedes and the internet” from 2021 which shows that people living in rural areas generally use e-services less, but when it comes to e-health services, the usage does not significantly differ to the overall Swedish population [23]. Furthermore, the existing SCB data shows no major changes in the distribution of the different groups since 2015. It therefore seems unlikely that the change indicated by the Tät data would be due to a distributional change in the overall Swedish population– even if the categories differ between Tät and SCB. The changes in the categories “rural area” and “district with fewer than 50,000 inhabitants” are both substantial in number and part of the same trend as the preceding years. The Tät study originated at a university located in the north of Sweden where there are large rural areas. Media reporting about the app and the research might have contributed to the proportionally large use in rural areas.

One strength of this study is the amount of data available from both Tät and SCB. Since the data was weighted, it enabled a comparison of the results without the study being confounded by age distribution relative to level of education and area of residence.

One weakness of this study is that the questionnaire is voluntary. This could entail a maldistribution of users answering the questionnaire versus real-world users. Another limitation is the comparison of Tät and SCB data, as the categories of the SCB data did not completely correspond with the categories in the Tät questionnaire, especially the area of residence. Furthermore, SCB had no data to provide for 2021 and 2022. This study used responses to a questionnaire presented to the users when they downloaded the app and does not take actual app-usage into account. The fact that before the update of the questionnaire in 2018 there was no information on gender or country of residence might have resulted in the inclusion of some men and some users from countries other than Sweden. However, the proportion of men using the app seems small. The proportion of users from other countries than Sweden before 2018 is more difficult to estimate but we have no reason to believe that this uncertainty would significantly change our conclusions about the educational level of the users.

Overall, the changes among Tät^®^ users indicate less disparity with the overall Swedish population. This is positive, although women with a university education are still over-represented when it comes to its usage. This is in line with a previous study showing that users of health apps were generally younger, had more education and higher income [24]. It is a great challenge for society and the healthcare system to identify possible reasons for this and to strive for change.

Sweden is a high-income country where smartphones and health apps are widely used. Our findings on how users of such apps have changed over time can probably best be generalized to similar contexts. However, the use of mobile health solutions is also rapidly increasing in lower income countries, and an app has the potential to reach a broad population [25].

## Conclusions

The proportion of Tät users in both the highest and lowest educational categories had changed to become more like the overall Swedish female population over the time period studied, although users with a high level of education were still over-represented. The proportion of Tät users who lived in rural areas had increased, and the results indicate major usage of the app among users in rural areas.

## Data Availability

The dataset analysed during the current study is available from the corresponding author on reasonable request.

## Abbreviations

UI: Urinary incontinence
SUI: Stress urinary incontinence
UUI: Urgency urinary incontinence
MUI: Mixed urinary incontinence
PFMT: Pelvic floor muscle training
RCT: Randomised controlled trial
SCB: Statistics Sweden
